# Supplementary material for: Rapid electron transfer by the carbon matrix in natural pyrogenic carbon
Source: Nat Commun. 2017 Mar 31;8:14873. doi: 10.1038/ncomms14873 (PMC5380966; doi:10.1038/ncomms14873)
Supplement: Supplementary Information — Supplementary Figures, Supplementary Tables, and Supplementary References [file ncomms14873-s1.pdf]

Supplementary Figures

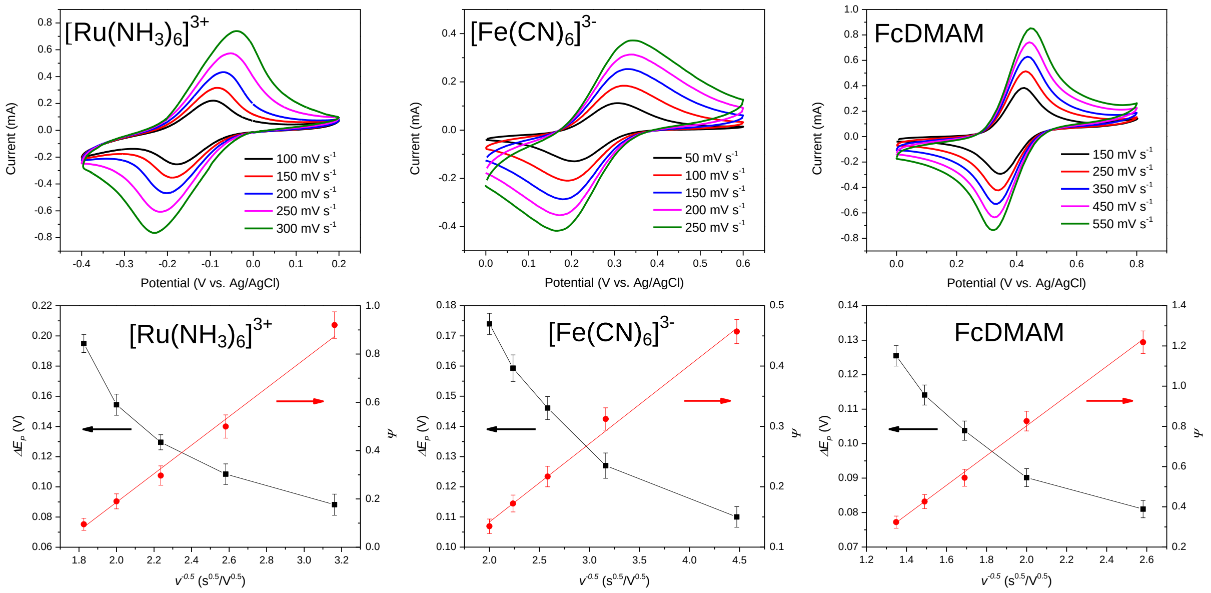

Supplementary Figure 1 | Redox couple selection for direct electron transfer of pyrogenic carbon matrices. Cyclic voltammograms of $[Ru(NH_3)_6]^{3+}$, $[Fe(CN)_6]^{3-}$ and FcDMAM (3 mM for all species in H_2O with 0.1 M KCl as the supporting electrolyte, pH=7) measured at a pyrogenic carbon (800°C) rod electrode using different potential scan rates listed in the chart legends (upper row). The Nicholson method (described in the Methods section) was used to calculate the electron transfer rate constant and the diffusion coefficients used in calculation were 6.5×10^{-6}, 7.6×10^{-6} and 5.1×10^{-6} for $[Ru(NH_3)_6]^{3+}$, $[Fe(CN)_6]^{3-}$ and FcDMAM, respectively[1-3]. Peak potential separation ΔE_P and Nicholson's kinetic parameter ψ versus the reciprocal of the square root of the potential scan rate $v^{-0.5}$ (lower row), based on which the heterogeneous electron transfer rate constants (k^0) were determined. Error bars are standard deviation of triplicate measurements.

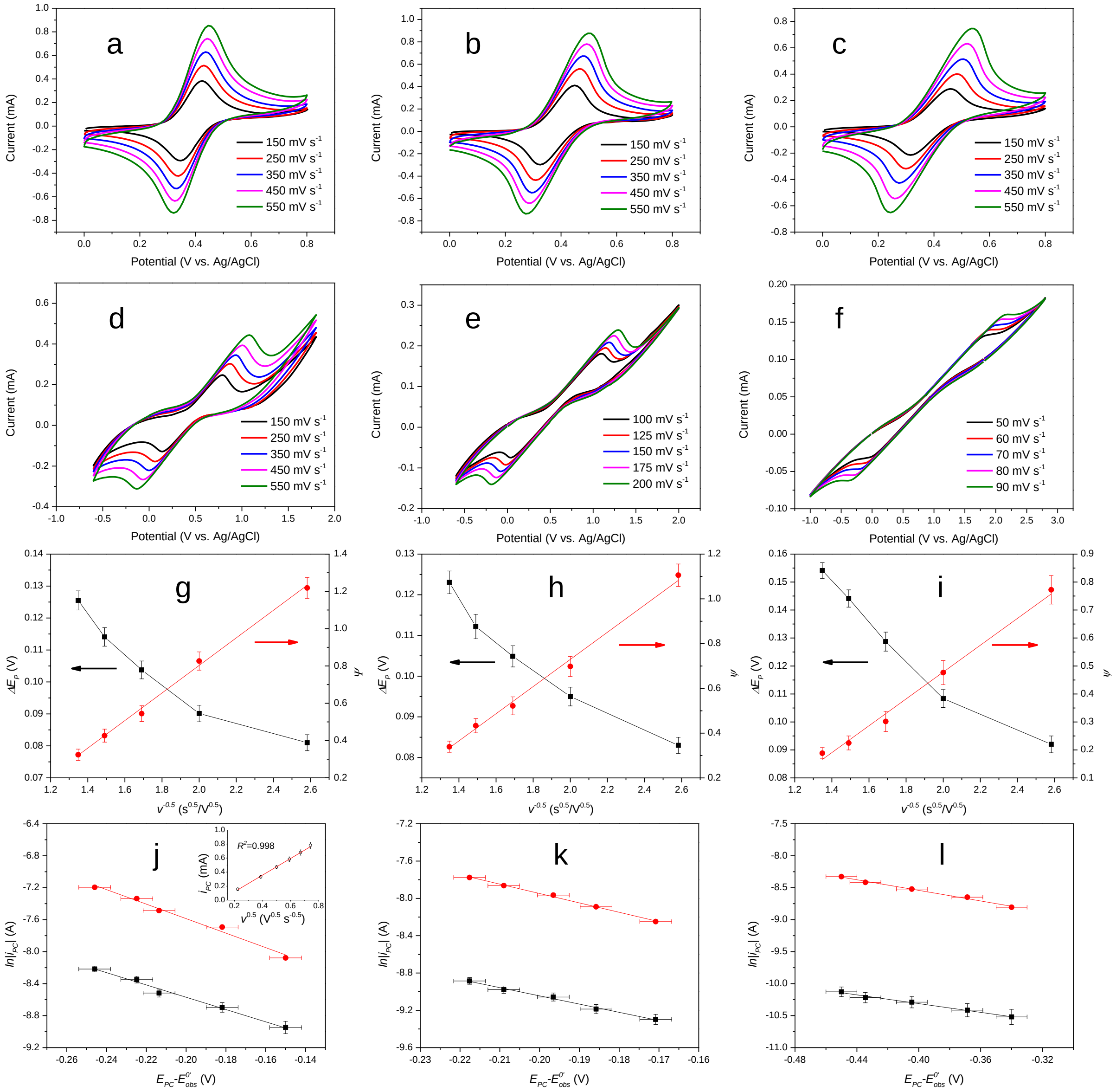

Supplementary Figure 2 | Electron transfer kinetics of pyrogenic carbon matrices pyrolysed at different temperatures. a-f, Cyclic voltammograms of FcDMAM (3 mM in H_2O with 0.1 M KCl as the supporting electrolyte, pH=7) measured at pyrogenic carbon rod electrodes pyrolysed at 800, 750, 725, 700, 675 and 650°C, respectively. Chart legends indicate different potential scan rates. **g-i,** Relationship of ΔE_P, $\Psi \sim v^{-1/2}$, from which the k^0 of pyrogenic carbon matrices 800, 750 and 725°C were calculated, respectively. **j-l,** Tafel plots used to calculate the k^0 of pyrogenic carbon matrices 700, 675 and 650°C, respectively. The red line indicates the upper limit and the black line the lower limit. Inset in **j** is the linear relationship of the peak current and the square root of the scan rate according to Randles–Sevcik equation, from which the upper limit current shown in **j** to **l** were calculated. Error bars are standard deviation of triplicate measurements.

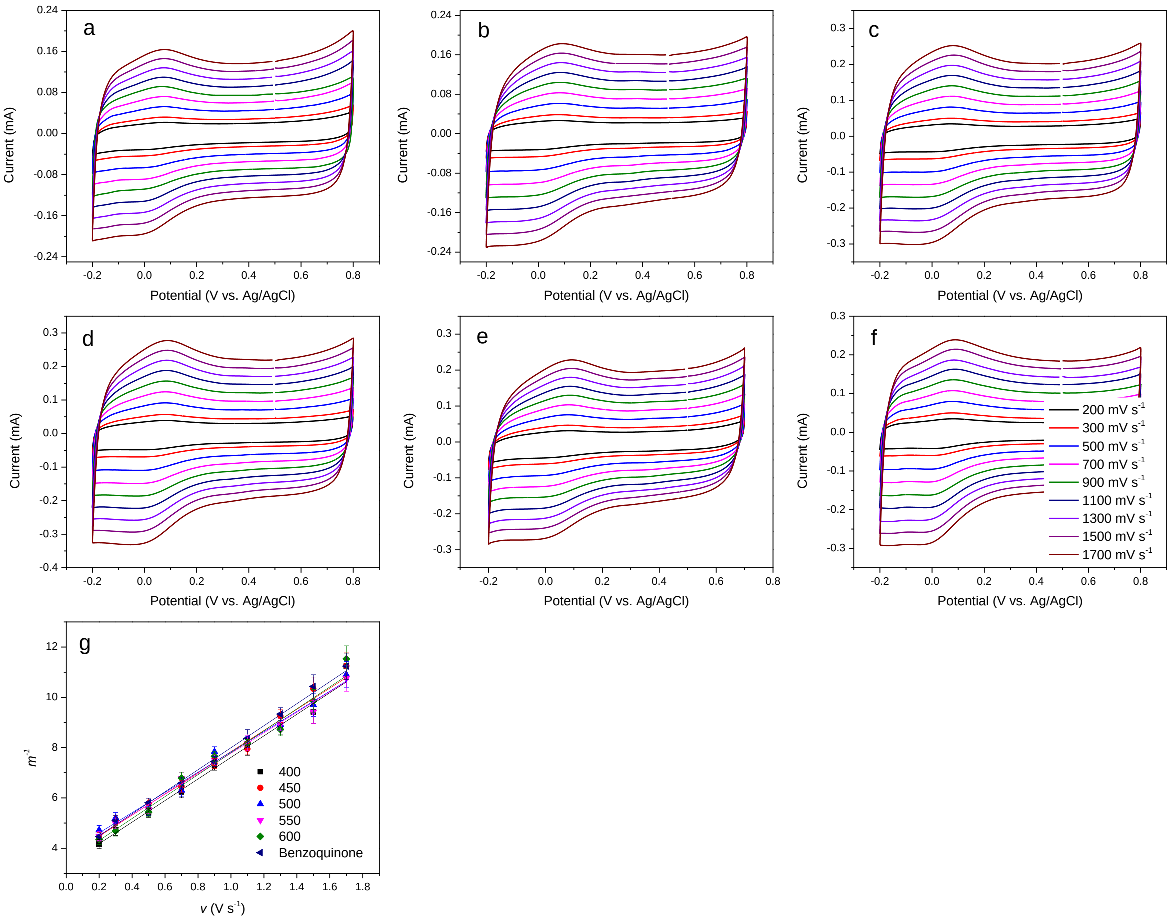

Supplementary Figure 3 | Electron charging and discharging kinetics of surface quinone groups of pyrogenic carbon. a-e, Immobilized cyclic voltammograms of surface quinone groups of pyrogenic carbon pyrolysed at 400, 450, 500, 550 and 600°C, respectively. Peak current decay and peak potential shift were observed for pyrogenic carbons produced at 400°C after several cycles of potential scans but not for pyrogenic carbons produced at higher temperature. This indicates that redox properties of surface quinone groups on low-temperature pyrogenic carbon is less stable than those on high-temperature pyrogenic carbon (data not shown). The pH values of all pyrogenic carbon samples were adjusted to 7 before immobilization on the graphite electrode. The supporting electrolyte was also buffered at pH 7 during the test since the cyclic voltammograms of surface quinone groups are pH-dependent. **f,** Immobilized cyclic voltammogram of benzoquinone (immobilization was done by recrystallization of 15 μL 0.2 mg mL^{-1} benzoquinone solution that contains 1.6μL Nafion). Chart legend in **f** indicates different potential scan rates and applied to **a-e**. **g,** Linear fits of the m^{-1}~v relationship which were used to calculate $k^{0'}$ of surface quinone groups and benzoquinone. Error bars are standard deviation of triplicate measurements.

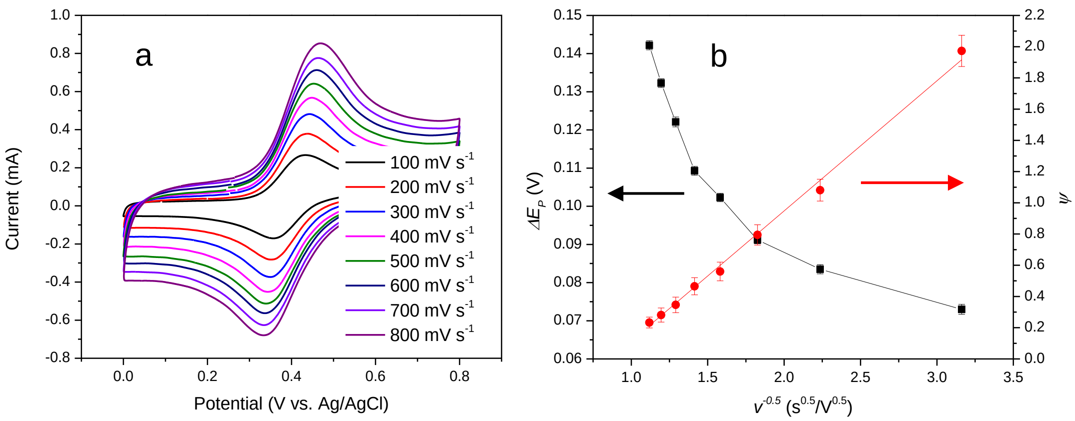

Supplementary Figure 4 | Theoretical maximum electron transfer kinetics of pyrogenic carbon matrices given by graphite. a, Cyclic voltammogram of FcDMAM (3 mM in H_2O with 0.1 M KCl as the supporting electrolyte, pH=7) measured at the graphite rod electrode. Chart legend indicates different potential scan rates. Graphite rods were purchased from Sigma-Aldrich (#496553, St. Louis, MO) and manufactured to 10 mm length and 5 mm diameter for the working electrodes. The electrode surfaces were carefully polished before measurement. **b,** Relationship of ΔE_P, $\Psi \sim v^{-1/2}$, from which the k^0 of graphite was calculated. Error bars are standard deviation of triplicate measurements.

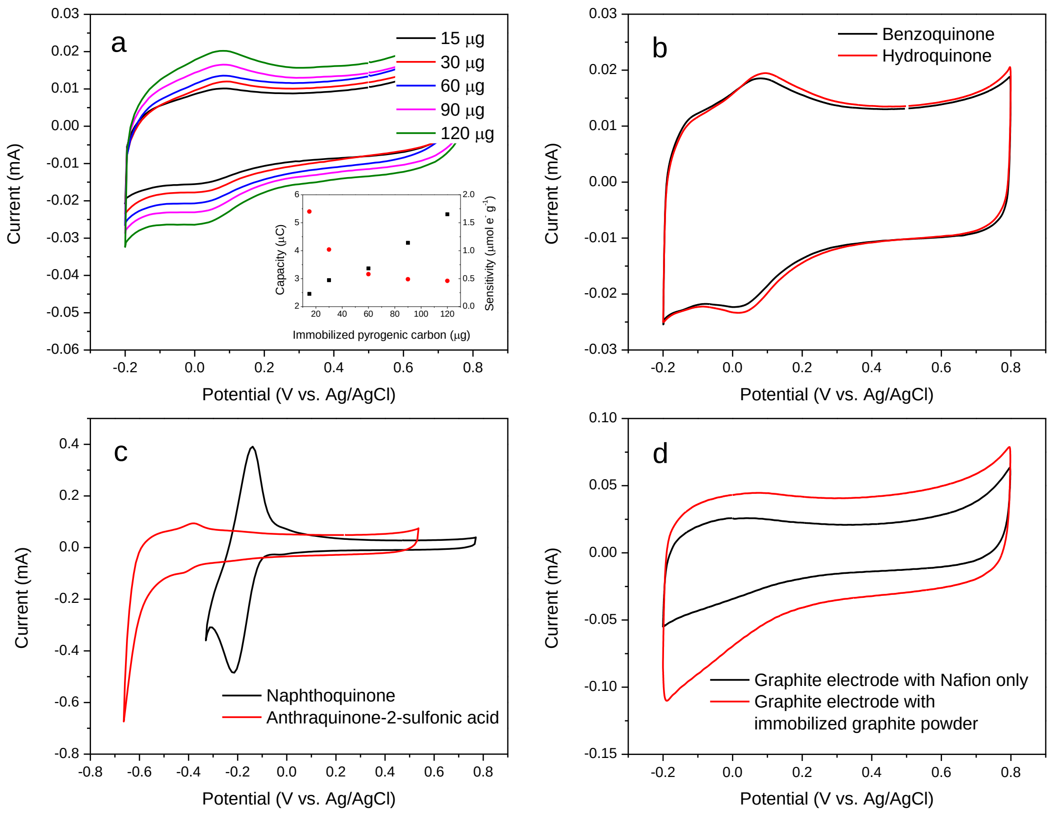

Supplementary Figure 5 | Immobilization of pyrogenic carbon and relevant quinone compounds on working electrode. a, Cyclic voltammogram of 550°C pyrogenic carbon (from low to high amounts of immobilized pyrogenic carbon as shown in the legend), **b,** benzoquinone and hydroquinone (3 µg), **c,** naphthoquinone and anthraquinone-2-sulfonic acid (3 µg), **d,** control tests on graphite working electrode with Nafion and immobilized graphite powder. Inset in **a** shows the total charging and discharging capacities (black dots) and sensitivities (red dots) as a function of the amount of immobilized pyrogenic carbon. Scan rate was 100 mV s^{-1}. Graphite rod (6mm diameter) was used as working electrode. pH of supporting electrolyte (0.1 M KCl) was buffered at 7 using phosphate buffering system.

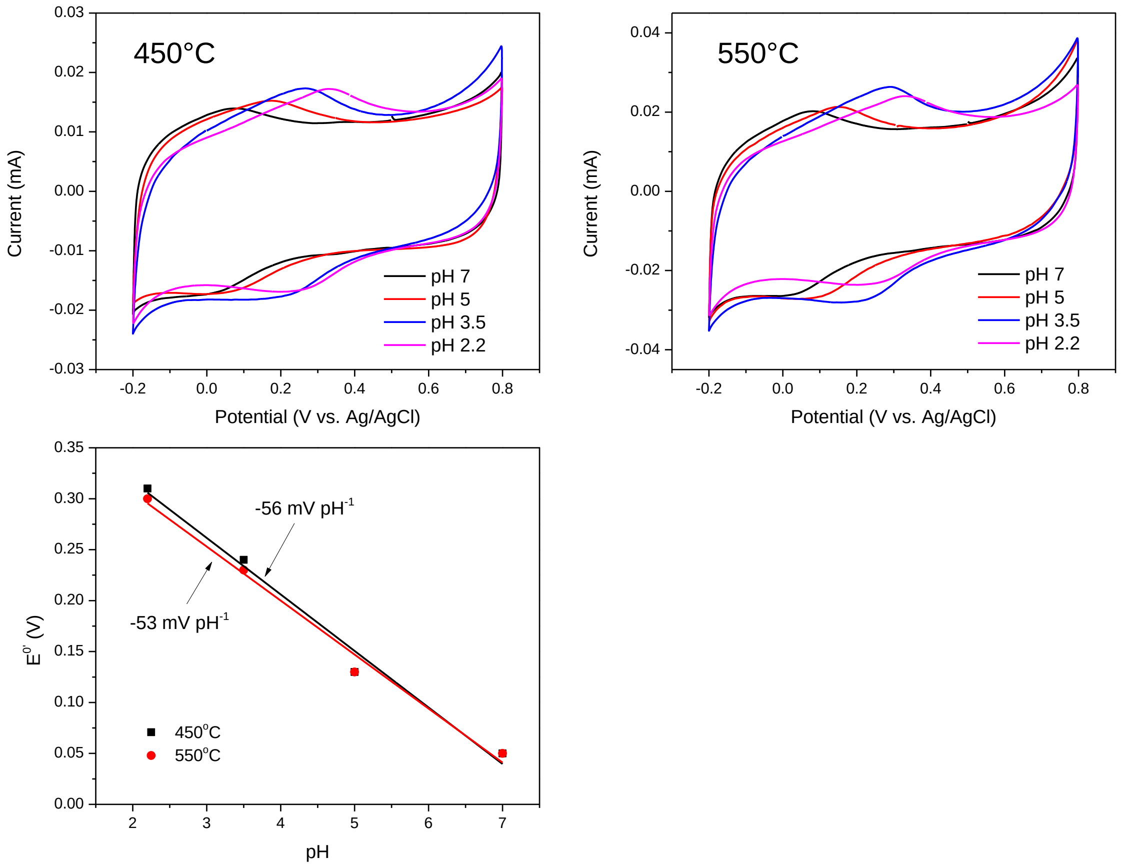

Supplementary Figure 6 | Dependence of formal potential $E^{0'}$ on pH for the surface quinone groups of pyrogenic carbon (pyrolysed at 450 and 550°C). Pyrogenic carbon powders were immobilized at a graphite working electrode, and voltammetric scan were performed in 0.1 M KCl solution at various pH values using a scan rate of 100 mV s^{-1}.

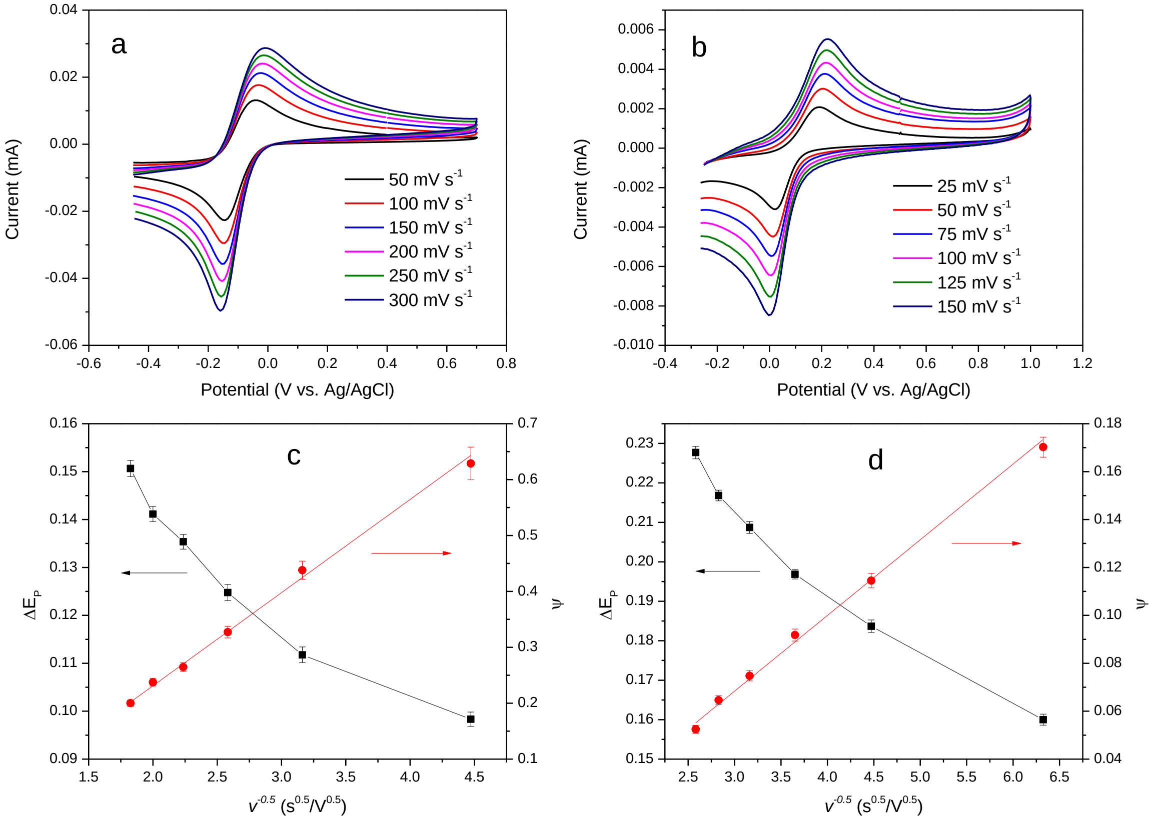

Supplementary Figure 7 | Estimation of k^0 of benzoquinone in solution. a and **b,** Cyclic voltammograms of 1, 4-benzoquinone in unbuffered and buffered solution, respectively. Chart legends indicate scan rates. **c** and **d,** Relationship of ΔE_P, $\Psi \sim v^{-1/2}$ following the Nicholson method, based on which k^0 values of benzoquinone in unbuffered and buffered solution were calculated, respectively. Error bars are standard deviation of triplicate measurements. pH of supporting electrolyte (0.1 M KCl) was 7 for both unbuffered and buffered solution. pH was buffered using a phosphate buffering system. pH adjustment in unbuffered solution was accomplished by potassium hydroxide and hydrogen chloride. Glassy carbon (3 mm diameter) was used as a working electrode. Estimated diffusion coefficients of benzoquinone for k^0 calculation were 4.1 and 7.6 × 10^{-6} cm^2 s^{-1} in unbuffered and buffered solution, respectively, which are in agreement with previously reported values[4,5].

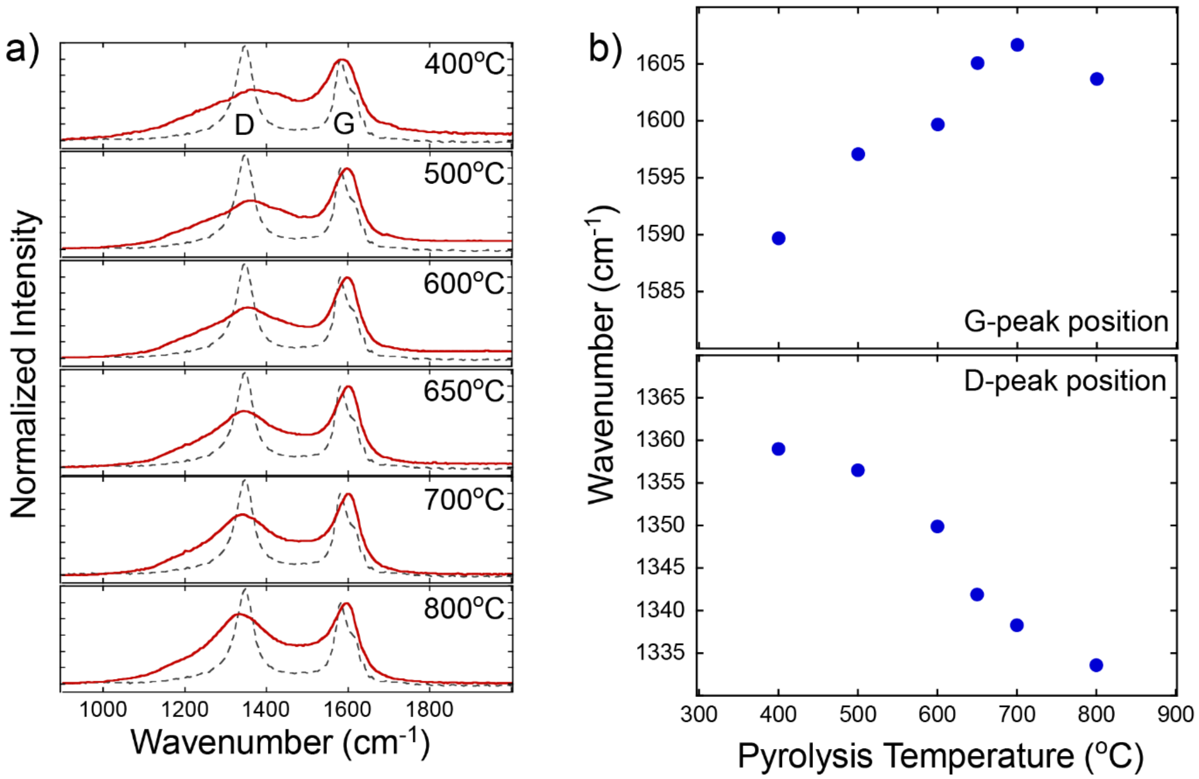

Supplementary Figure 8 | a, Averaged Raman spectra from areas of a finely polished pyrogenic carbon at different pyrolysis temperatures. The Raman spectrum from a sample of commercial graphite (Sigma Aldrich) is shown for comparison. **b,** Positions of the *G* peak and *D* peak in the Raman spectra at different pyrolysis temperatures, obtained by fitting a Lorentzian curve to the *D* peak, and a Breit-Wigner-Fano curve to the *G* peak. The *G* peak was observed to shift to higher wavenumber between 400°C and 700°C, followed by a small shift to lower wavenumber at 800°C. The *D* peak was observed to shift to lower wavenumber as pyrolysis temperature increased. We note that a similar trend in the *D* peak position occurs in coal. The *D* peak of lignite coal, which has relatively low carbon content, and relatively high impurity content, is at a higher wavenumber than that of anthracite, which has relatively high carbon content, and low impurity[6]. Similar trends in the position of the *D* and *G* peaks have also been observed in carbon films annealed at different temperatures[7].

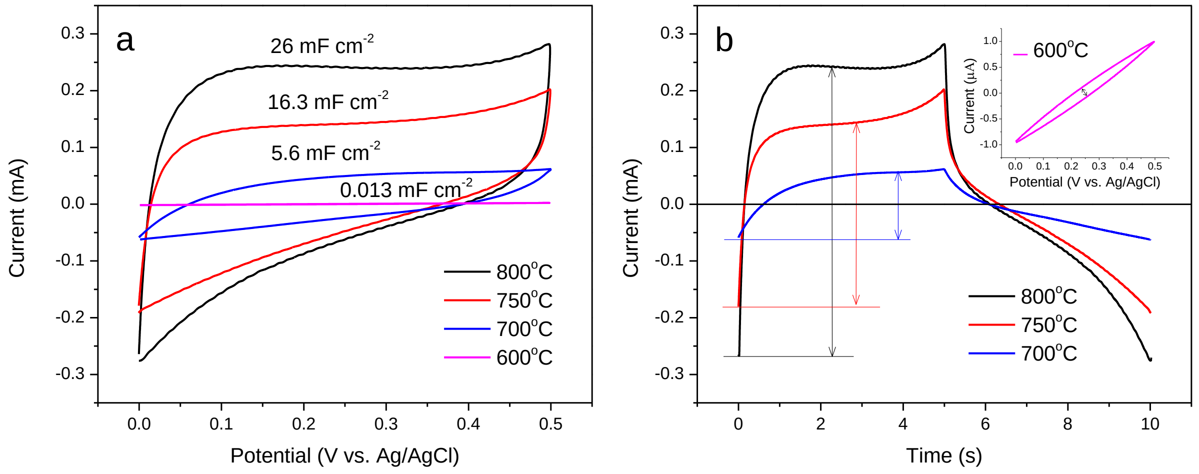

Supplementary Figure 9 | Determination of electrochemical capacitance of pyrogenic carbon. a, Cyclic voltammograms of pyrogenic carbon in 0.1 M KCl at a scan rate of 100 mV s^{-1}. **b,** Current-time plot derived from **a** for determination of the capacitive current (shown by arrows).

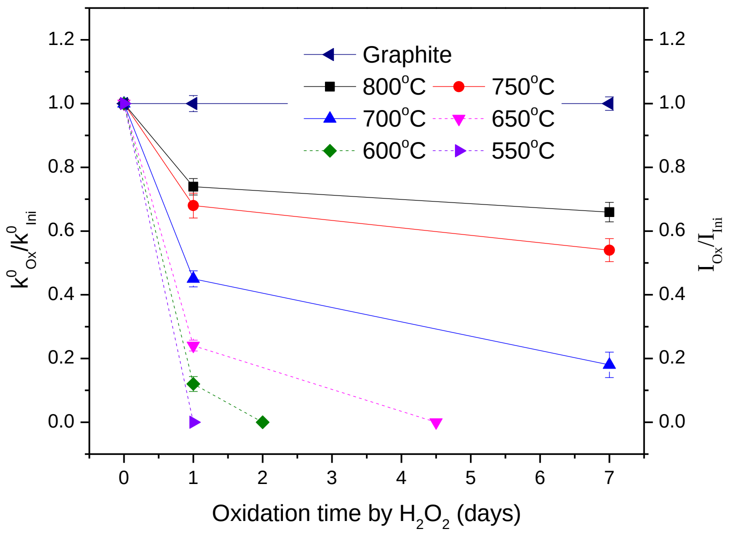

Supplementary Figure 10 | Prediction of natural aging by H_2O_2 oxidation. Effect of aging processes on electron transfer kinetics of pyrogenic carbon matrices. Ratios of k^0 between oxidized and initial pyrogenic carbon (solid lines) were calculated based on cyclic voltammograms in Supplementary Fig. 15. The current (I) ratio (dashed lines) was calculated based on chronoamperograms in Supplementary Fig. 16. Error bars are standard deviation of triplicate measurements.

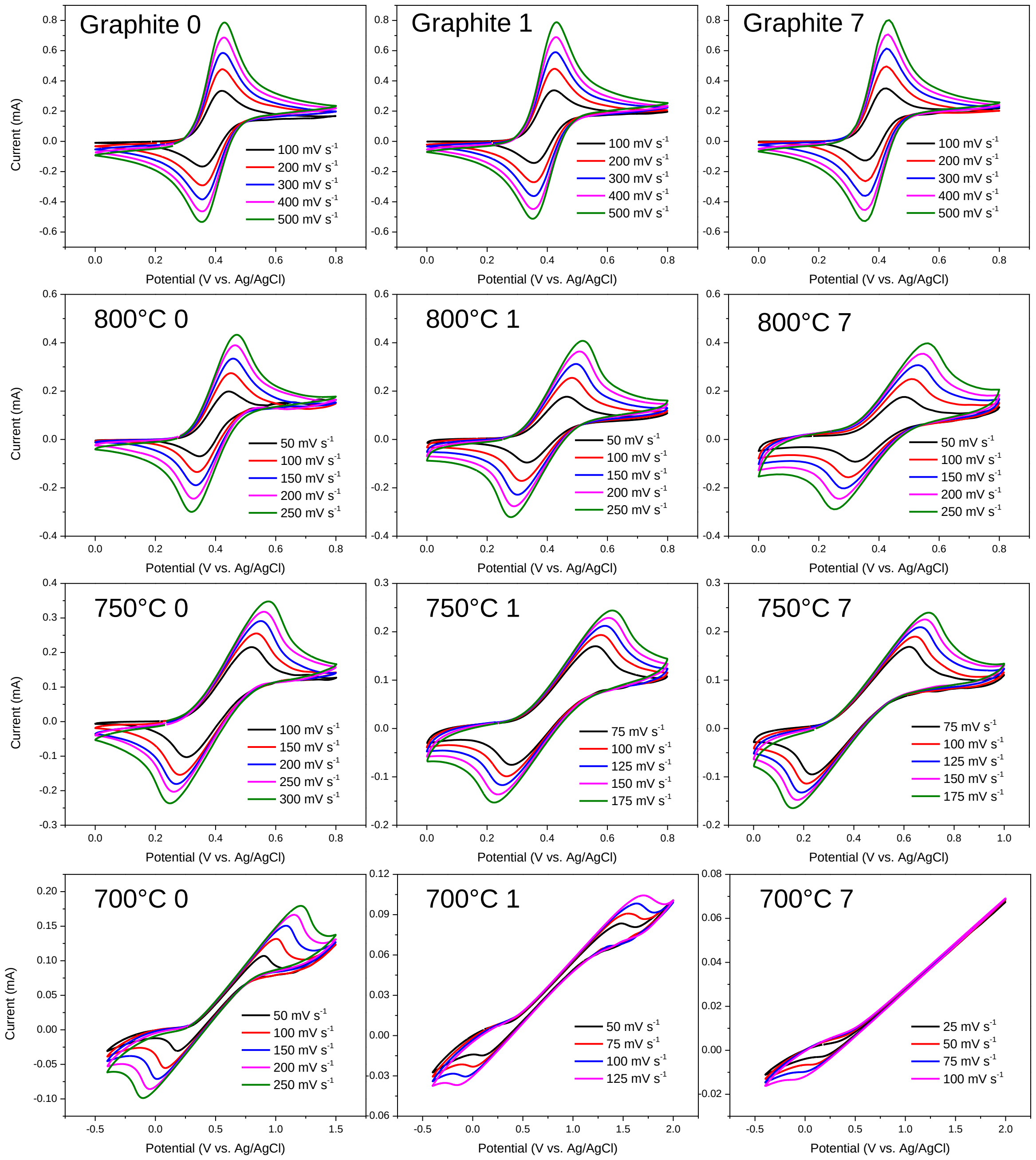

Supplementary Figure 11 | Cyclic voltammograms of graphite and pyrogenic carbon 800, 750 and 700°C after 0, 1 and 7 days of oxidation with H_2O_2. Cyclic voltammetry measurements were performed with FcDMAM (3 mM in H_2O with 0.1 M KCl as supporting electrolyte, pH=7) at graphite or pyrogenic carbon paste electrodes. pH values of all pyrogenic carbons were adjusted to the initial value after oxidation. Chart legends indicate different potential scan rates.

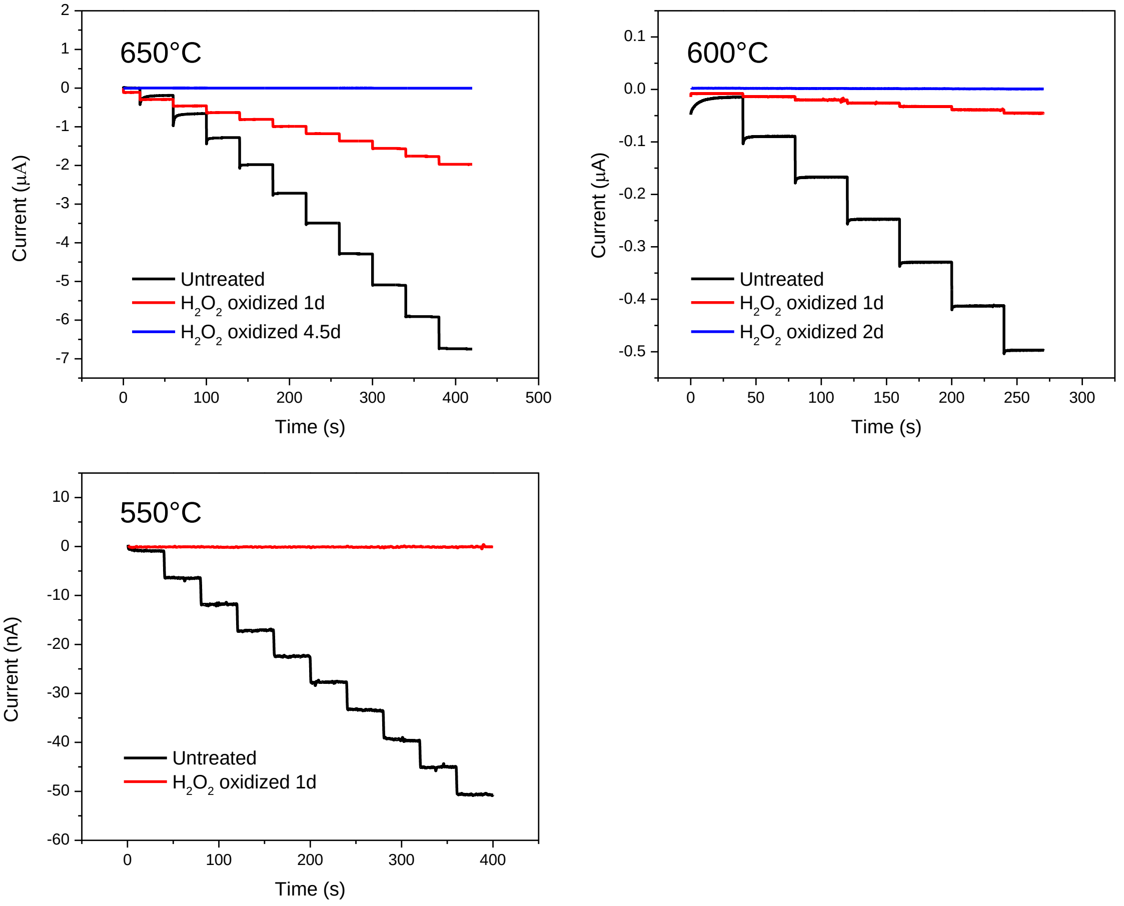

Supplementary Figure 12 | Chronoamperograms of pyrogenic carbons produced at 650, 600 and 550°C before and after H_2O_2 oxidation. The applied potential was -0.2 V for every step shown in chronoamperograms. Passing current is identified by the current difference between steps. Chronoamperometry measurements were carried out in H_2O with 0.1 M KCl as the supporting electrolyte, pH=7 by pyrogenic carbon paste electrodes.

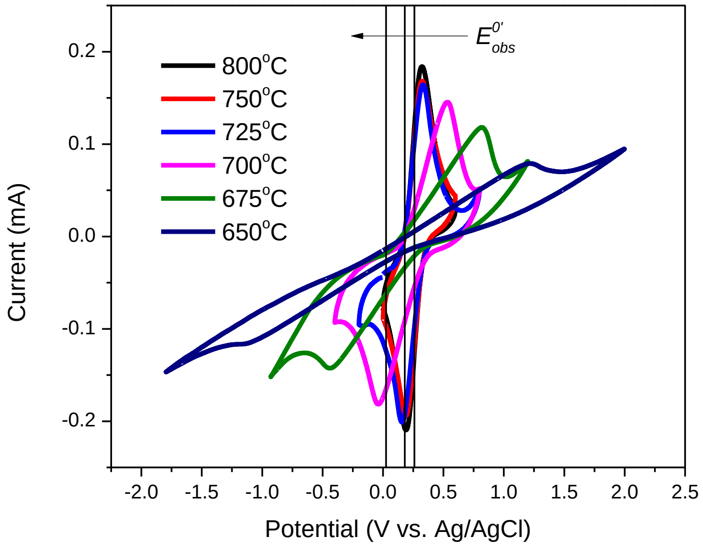

Supplementary Figure 13 | Inner resistance induced formal potential deviation. Cyclic voltammograms were $[Fe(CN)_6]^{3-}$ (3 mM in H_2O with 0.1 M KCl as the supporting electrolyte, pH=7) at pyrogenic carbon working electrodes. The scan rate for pyrogenic carbon 800 to 675°C was 150 mV s^{-1} and 55 mV s^{-1} for pyrogenic carbon 650°C.

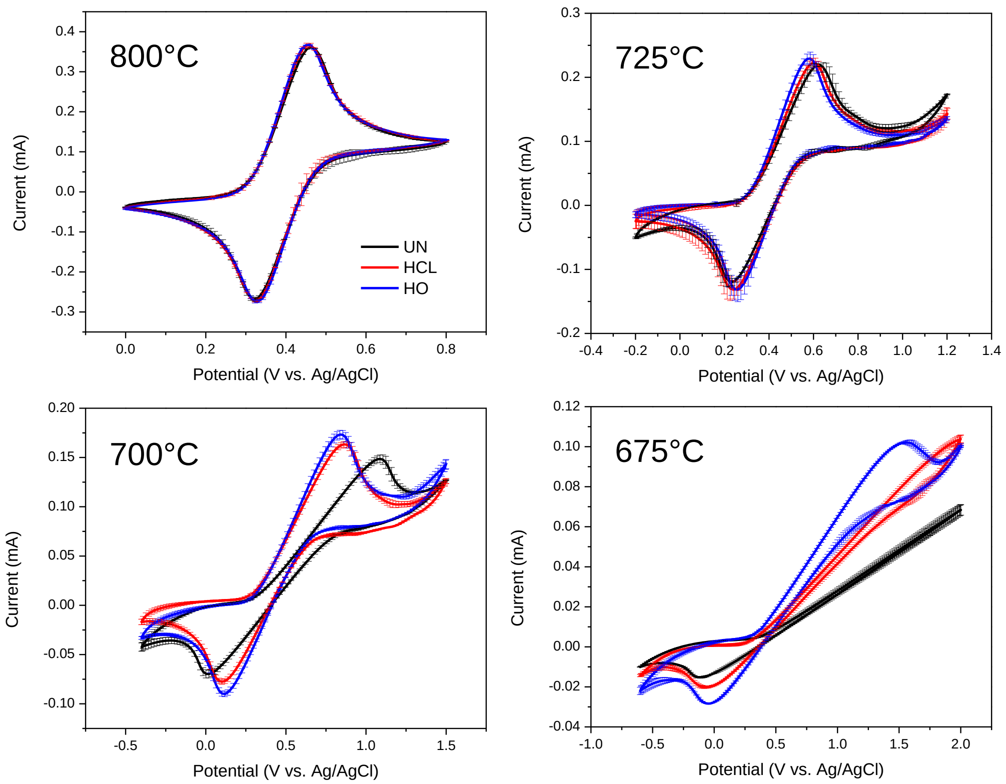

Supplementary Figure 14 | Influence of inner resistance on electron transfer of pyrogenic carbon matrices. UN is untreated pyrogenic carbon, HCL indicates the pyrogenic carbon washed with hydrogen chloride (0.1 M, shaking overnight) and HO indicates the pyrogenic carbon washed by hydrogen chloride and organic solvents (sequentially, ethanol, acetone, chloroform and toluene, shaking overnight). pH of all pyrogenic carbons were adjusted to initial values after washing. Cyclic voltammograms were obtained using FcDMAM (3 mM in H_2O with 0.1 M KCl as supporting electrolyte, pH=7) on pyrogenic carbon paste electrodes after different treatments. The scan rate was 150 mV s^{-1} for all measurements. Error bars are standard deviation of triplicate measurements.

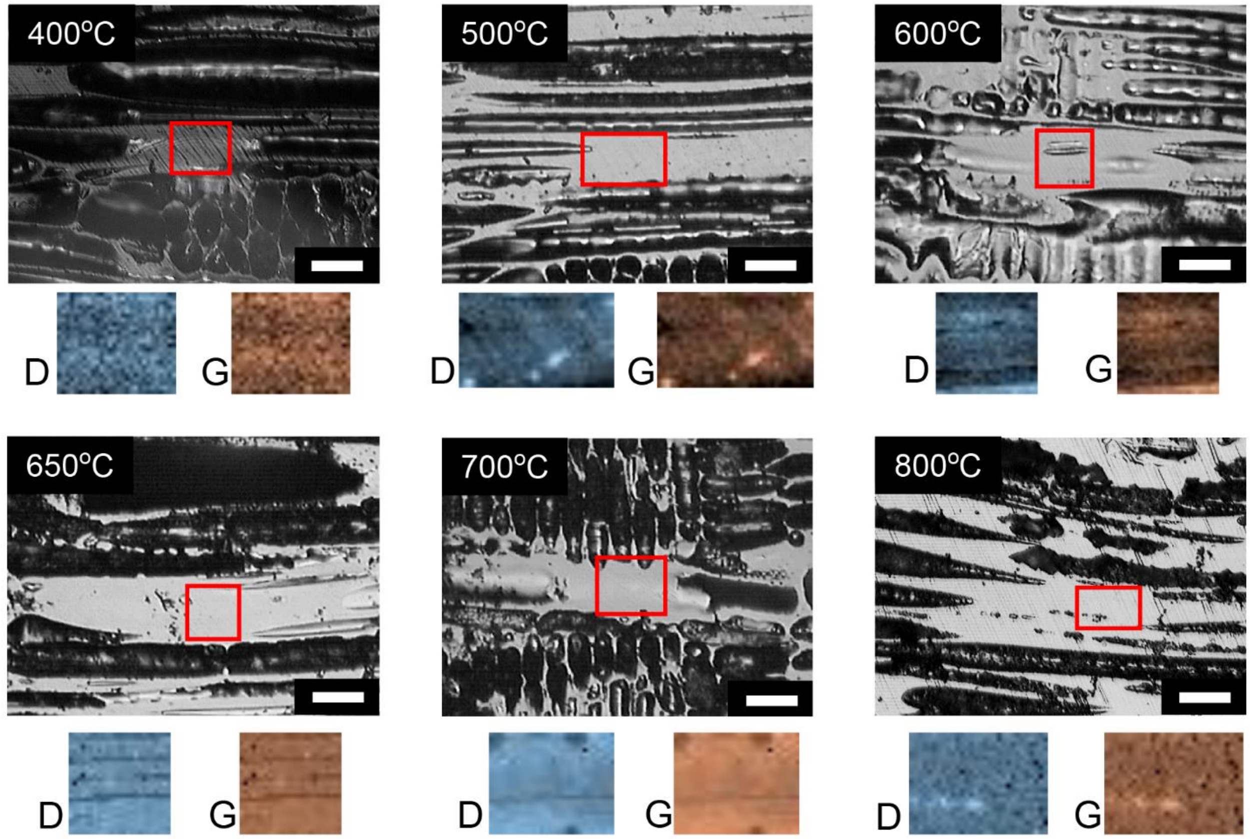

Supplementary Figure 15 | Optical images of polished surfaces of pyrogenic carbon samples. Raman spatial maps were acquired from the area indicated by a red box on the image. These areas were selected for Raman spatial mapping because they were observed to be optically flat. Maps of the *D* and *G* peak intensities show no features unique to either peak, suggesting that the structural ordering of the carbon in the pyrogenic carbon samples was homogeneous over the areas mapped, at 1 µm length scale. Scale bars 25 µm.

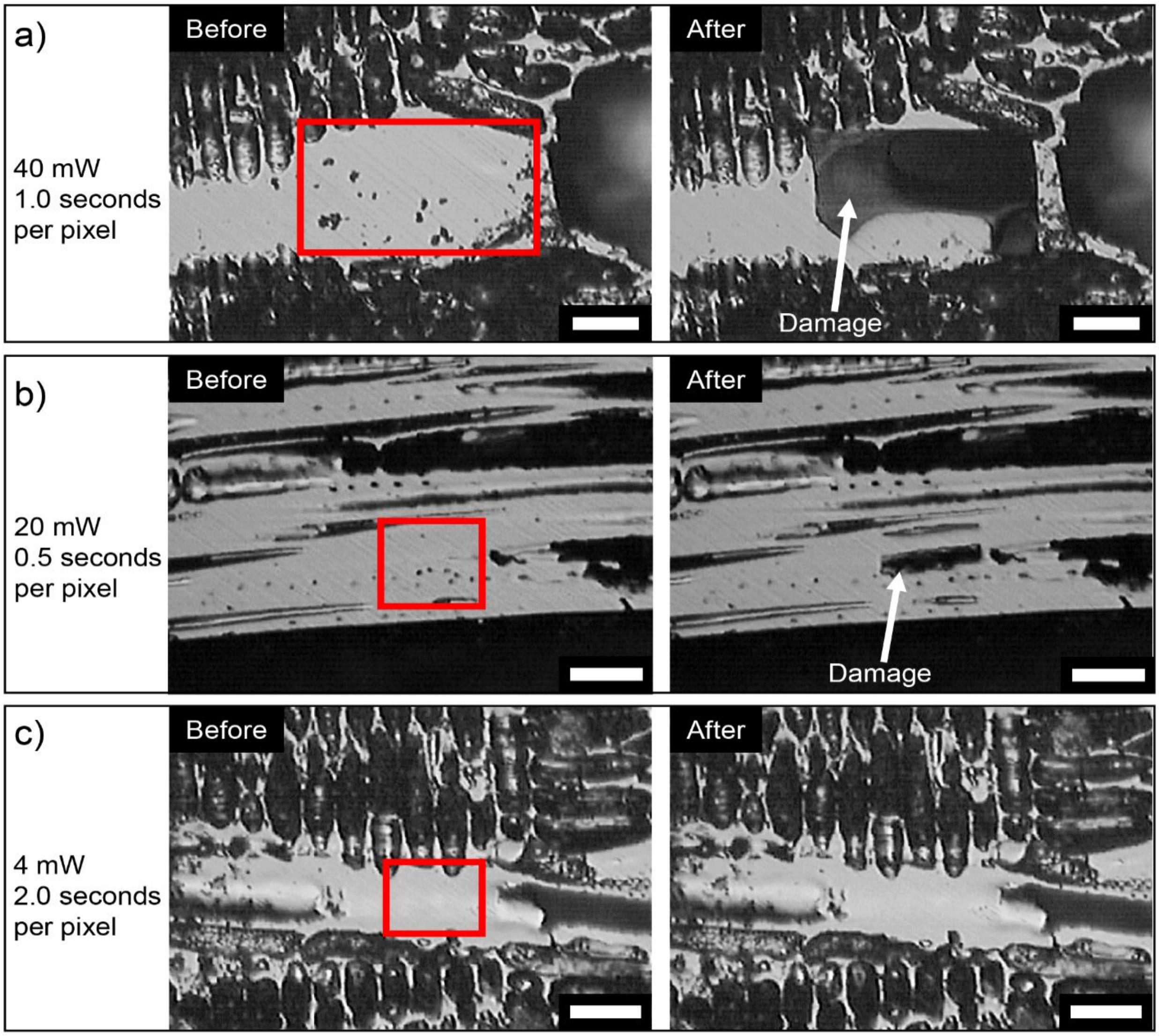

Supplementary Figure 16 | Optical images of a sample of pyrogenic carbon, pyrolysed at 700°C. **a,** A region of the sample before and after acquisition of Raman spectroscopic maps with a 532 nm laser at 40 mW power, with a dwell time of 1 second per pixel. After acquisition severe damage to the surface of the sample is clearly visible in the optical microscope. This suggests that the structure of the carbon in the sample was altered by the laser during acquisition, rendering the resulting spectra unreliable. **b,** A region of the sample before and after acquisition of Raman spectroscopic maps with a 532 nm laser at 20 mW power, with a dwell time of 0.5 seconds per pixel. Damage is more moderate, but still occurs. **c,** A region of the sample before and after acquisition of Raman spectroscopic maps with a 532 nm laser at 4 mW power, with a dwell time of 2 seconds per pixel. In this case, optical images show no significant damage to the surface of the sample, suggesting that carbon structure was not significantly altered by the laser, and therefore that the Raman spectra obtained would be reliable. Scale bars 25 µm.

Supplementary Tables

Supplementary Table 1 | Elemental composition analysis of the pyrogenic carbon samples from low to high pyrolysis temperature.

Pyrogenic carbon sample	Total elemental contents (% w/w)				Molar ratio		pH[a]
	C	N	H	O	O/C	H/C	
400°C	74.21	0.56	3.86	23.92	0.24	0.62	4.39
450°C	82.40	0.66	3.50	17.22	0.16	0.51	7.85
500°C	78.95	0.68	3.19	17.01	0.16	0.49	7.90
550°C	83.07	0.68	2.54	11.23	0.10	0.37	8.45
600°C	86.03	0.65	2.50	10.78	0.09	0.35	8.53
650°C	89.03	0.71	1.97	8.91	0.08	0.27	8.57
700°C	88.67	0.66	1.43	7.63	0.06	0.19	8.82
800°C	91.45	0.70	0.80	5.69	0.05	0.11	9.23

[a] pH measured in 1M KCl solution.

Supplementary Table 2 | Comparison of formal potentials between surface quinone of pyrogenic carbon and model quinone compounds.

Quinone compound	Chemical structure	Formal potential $E^{0'}$ (V vs. SHE)	pH*	Type of cyclic voltammetry	Reference
Hydroquinone	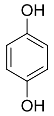	0.344	7	Diffusive	Ref.[8]
		0.265	7	Immobilized	This study
Benzoquinone	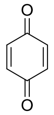	0.324	6	Immobilized	Ref.[9]
		0.25	7	Immobilized	This study
		0.199	7	Immobilized	Ref.[10]
Surface functional group of carbon black		0.344	7	Immobilized	Ref.[11]
PVHQ	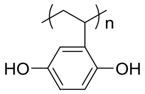	0.279	7	Immobilized	Ref.[12]
Surface quinone of pyrogenic carbon		0.25	7	Immobilized	This study
Ubiquinone disulfides	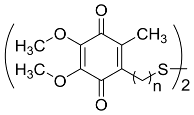	0.044 – 0.119	7	Immobilized	Ref.[13]
Ubiquinone Q_{10}	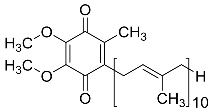	0.074	7	Immobilized	Ref.[14]
Naphthoquinone and derivatives	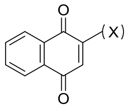	-0.109 – 0.121	7	Immobilized	Ref.[15,16]
		0.019	7	Immobilized	This study
AQDS	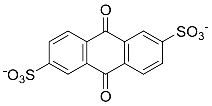	-0.156	7	Diffusive	Ref.[17]
		-0.18	7	Diffusive	Ref.[18]
Riboflavin	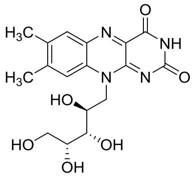	-0.2	7	Immobilized	Ref.[19]
		-0.22	7	Diffusive	Ref.[18]
AQS	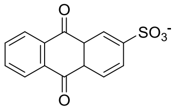	-0.2	7	Immobilized	This study
		-0.24	7	Diffusive	Ref.[18]
Surface quinone of soil organic matter		0.15 – -0.3	7	Mediated chronoamperometry	Ref.[18,20]

*pH is buffered using a phosphate buffering system.

Supplementary Table 3 | Summarization of techniques, operational conditions, and purposes of the electrochemical test performed in this study.

Technique	Working electrode	Redox couple[a]	Binding reagent[b]	Supporting electrolyte[c]	Purpose
Cyclic voltammetry	Pyrogenic carbon rod electrode	In dissolved phase, $[Ru(NH_3)_6]^{3+/2+}$ $([Fe(CN)_6]^{3-/4-}$ $FcDMAM^{+/0}$	N/A	0.1 M KCl at pH 7	Testing the direct electron transfer kinetics of pyrogenic carbon matrices
Cyclic voltammetry	Graphite rod electrode	In immobilized phase, pyrogenic carbon powder pyrolysed from 400 to 800°C BO, HO, NO, ADS	Nafion	0.1 M KCl at pH 7	Testing the charging and discharging kinetics of surface functional groups on pyrogenic carbon
Cyclic voltammetry	Pyrogenic carbon rod electrode	N/A	N/A	0.1 M KCl at pH 7	Determination of electrochemical capacitance of pyrogenic carbon
Cyclic voltammetry	Pyrogenic carbon paste electrode	In dissolved phase, $FcDMAM^{+/0}$	N/A	0.1 M KCl at pH 7	Investigating the effect of minerals and easily mineralizable organic matter on electron transfer of pyrogenic carbon matrices
Linear sweep voltammetry	Pyrogenic carbon rod pyrolysed at 800°C	In immobilized phase, MnO_2 $FeCl_3$ magnetite, hematite	Nafion	0.1 M KCl at pH 7	Characterizing the potential range of pyrogenic carbon on transferring electrons to various minerals
Cyclic voltammetry and chronoamperometry	Pyrogenic carbon paste electrode	In dissolved phase, $FcDMAM^{+/0}$	N/A	0.1 M KCl at pH 7	Investigating the effect of natural aging on electron transfer of pyrogenic carbon matrices

[a] All redox couples were tested separately. [b] Binding reagent was applied only on immobilized redox couple. [c] pH of supporting electrolyte was buffered by phosphate buffering system.

Supplementary Table 4 | Inner resistances of pyrogenic carbon rod electrodes (ohm) at different pyrolysis temperatures (°C).

800	750	725	700	675	650
7±0.17	40±4.9	110±22	1300±153	4200±525	18000±971

Note: All resistances were measured by a multimeter immediately prior to performing the electrochemical tests. Aluminum foil was clamped on both ends of the electrode during measurement to improve the contact between electrodes and the multimeter probes and prevent potential damaging caused by direct contact with the probe.

Supplementary Table 5 | Peak assignment for C forms obtained from FTIR and EELS spectroscopy. Attenuated total reflectance Fourier transform infrared (ATR-FTIR) spectroscopy and electron energy loss spectroscopy (EELS) were used to investigate the transition of surface functionalities of pyrogenic carbon from low to high pyrolysis temperature. Peak assignment of FTIR spectra was done according to ref[21-23], and assignment of EELS spectra was done according to ref[21,24-27].

Energy Level	Carbon Forms
	FTIR, cm^{-1}
3620	O-H stretching vibrations of alcoholic and phenolic -OH, not hydrogen bonded
3320	O-H stretching vibrations of hydrogen bonded hydroxyl (-OH) groups
3010	C-H stretching of aromatic-C
2910-2850	weak C-H stretching vibrations of aliphatic-C
1720-1690	C=O stretching mainly of carboxyl-C and traces of ketones and esters
1590	C=C stretching of aromatic-C and C=O stretching of conjugated ketones and quinones
1500-1140	C=C stretching of aromatic skeletal vibrations, indicative of lignin, in plane O-H bending of phenolic -OH, and C-O stretching of ester groups in cellulose and hemicellulose
1020	C-O stretching vibrations of aliphatic-C
880-750	aromatic C-H out-of-plane deformation
	EELS (carbon k-edge), eV
284.1	quinone C=C
284.9	aromatic and graphitic C=C
286.1	olefin and heteroaromatic C=O
287.4	aliphatic C-H and phenolic C-OH
289	carbonyl-C, alcohol C-O
291.9	aromatic C-C

Supplementary Table 6 | Polishing procedure for pyrogenic carbon by diamond sand paper.

Step	Grain size on sand paper (µm)	Rotation speed (rpm)	Removed depth (µm)
1	15	100	100
2	6	70	500
3	3	50	20
4	1	50	10-15

Supplementary References

1 Velmurugan, J., Zhan, D. & Mirkin, M. V. Electrochemistry through glass. *Nat. Chem.* **2**, 498-502 (2010).

2 Bard, A. J. & Faulkner, L. R. *Electrochemical Methods: Fundamentals and Applications* (Wiley, 2001).

3 Nakatani, K., Wakabayashi, M., Chikama, K. & Kitamura, N. Electrochemical studies on mass transfer of ferrocene derivatives across a single-nitrobenzene-microdroplet/water interface. *J. Phys. Chem.* **100**, 6749-6754 (1996).

4 Babanova, S., Matanovic, I., Chavez, M. S. & Atanassov, P. Role of quinones in electron transfer of pqq–glucose dehydrogenase anodes—mediation or orientation effect. *J. Am. Chem. Soc.* **137**, 7754-7762 (2015).

5 Ji, X. *et al.* Electrode kinetic studies of the hydroquinone−benzoquinone system and the reaction between hydroquinone and ammonia in propylene carbonate: Application to the indirect electroanalytical sensing of ammonia. *J. Phys. Chem. C* **111**, 1496-1504 (2007).

6 Makovsky, L. E., Waldstein, P. & Edwards, W. H. Raman spectra of coals. *Nat. Phys. Sci.* **231**, 154-155 (1971).

7 Dillon, R. O., Woollam, J. A. & Katkanant, V. Use of raman scattering to investigate disorder and crystallite formation in as-deposited and annealed carbon films. *Phys. Rev. B* **29**, 3482-3489 (1984).

8 Liu, X. *et al.* Electrochemical behavior of hydroquinone at multi-walled carbon nanotubes and ionic liquid composite film modified electrode. *Colloids Surf. B* **79**, 27-32 (2010).

9 Aklilu, M., Tessema, M. & Redi-Abshiro, M. Indirect voltammetric determination of caffeine content in coffee using 1,4-benzoquinone modified carbon paste electrode. *Talanta* **76**, 742-746 (2008).

10 Rosendahl, S. M. & Burgess, I. J. Charge transfer and seiras studies of 1,4-benzoquinone functionalized mixed monothiol/dithiol self-assembled monolayers. *Electrochim. Acta* **56**, 4361-4368 (2011).

11 Jow, J.-J. *et al.* Determination of surface area of carbon-black by simple cyclic-voltammetry measurements in aqueous H_2SO_4. *J. Ind. Eng. Chem.* **19**, 1730-1734 (2013).

12 Takada, K., Gopalan, P., Ober, C. K. & Abruña, H. D. Synthesis, characterization, and redox reactivity of novel quinone-containing polymer. *Chem. Mater.* **13**, 2928-2932 (2001).

13 Ma, W. *et al.* Investigating electron-transfer processes using a biomimetic hybrid bilayer membrane system. *Nat. Protoc.* **8**, 439-450 (2013).

14 Takehara, K. & Ide, Y. Electrochemical behaviour of the ubiquinone-Q_{10} film coated onto a glassy carbon electrode by the spinner method. *Bioelectroch. Bioener.* **26**, 297-305 (1991).

15 Milton, R. D. *et al.* Rational design of quinones for high power density biofuel cells. *Chem. Sci.* **6**, 4867-4875 (2015).

16 Golabi, S. M. & Raoof, J. B. Catalysis of dioxygen reduction to hydrogen peroxide at the surface of carbon paste electrodes modified by 1,4-naphthoquinone and some of its derivatives. *J. Electroanal. Chem.* **416**, 75-82 (1996).

17 Batchelor-McAuley, C., Li, Q., Dapin, S. M. & Compton, R. G. Voltammetric characterization of DNA intercalators across the full pH range: Anthraquinone-2,6-disulfonate and anthraquinone-2-sulfonate. *J. Phys. Chem. B* **114**, 4094-4100 (2010).
18 Klüpfel, L., Piepenbrock, A., Kappler, A. & Sander, M. Humic substances as fully regenerable electron acceptors in recurrently anoxic environments. *Nat. Geosci.* **7**, 195-200 (2014).
19 Sun, W., Kong, J., Deng & Jiaqi. Electrocatalytic reduction of hemoglobin at a chemically modified electrode containing riboflavin. *Electroanalysis* **9**, 115-119 (1997).
20 Aeschbacher, M., Vergari, D., Schwarzenbach, R. P. & Sander, M. Electrochemical analysis of proton and electron transfer equilibria of the reducible moieties in humic acids. *Environ. Sci. Technol.* **45**, 8385-8394 (2011).
21 Keiluweit, M., Nico, P. S., Johnson, M. G. & Kleber, M. Dynamic molecular structure of plant biomass-derived black carbon (biochar). *Environ. Sci. Technol.* **44**, 1247-1253 (2010).
22 Jindo, K. *et al.* Physical and chemical characterization of biochars derived from different agricultural residues. *Biogeosciences* **11**, 6613-6621 (2014).
23 Lehmann, J. *et al.* Near-edge X-ray absorption fine structure (NEXAFS) spectroscopy for mapping nano-scale distribution of organic carbon forms in soil: Application to black carbon particles. *Global Biogeochem. Cycles* **19**, GB1013 (2005).
24 Francis, J. T. & Hitchcock, A. P. Inner-shell spectroscopy of p-benzoquinone, hydroquinone, and phenol: Distinguishing quinoid and benzenoid structures. *J. Phys. Chem.* **96**, 6598-6610 (1992).
25 Solomon, D. *et al.* Carbon (1s) NEXAFS spectroscopy of biogeochemically relevant reference organic compounds *Soil Sci. Soc. Am. J.* **73**, 1817-1830 (2009).
26 Braun, A., Kubatova, A., Wirick, S. & Mun, S. B. Radiation damage from EELS and NEXAFS in diesel soot and diesel soot extracts. *J. Electron. Spectrosc. Relat. Phenom.* **170**, 42-48 (2009).
27 Cody, G. D. *et al.* Determination of chemical-structural changes in vitrinite accompanying luminescence alteration using C-NEXAFS analysis. *Org. Geochem.* **28**, 441-455 (1998).
